# Tumor-Infiltrating iNKT Cells Activated through c-Kit/Sca-1 Are Induced by Pentoxifylline, Norcantharidin, and Their Mixtures for Killing Murine Melanoma Cells

**DOI:** 10.3390/ph16101472

**Published:** 2023-10-16

**Authors:** Maximiliano V. M. Correa-Lara, Israel Lara-Vega, Minerva Nájera-Martínez, María Lilia Domínguez-López, Elba Reyes-Maldonado, Armando Vega-López

**Affiliations:** 1 Laboratorio de Toxicología Ambiental, Escuela Nacional de Ciencias Biológicas, Instituto Politécnico Nacional, Av. Wilfrido Massieu s/n, Unidad Profesional Zacatenco, Mexico City CP 07738, Mexiconajmtz@yahoo.com.mx (M.N.-M.); 2 Laboratorio de Inmunoquímica I, Escuela Nacional de Ciencias Biológicas, Instituto Politécnico Nacional, Carpio y Plan de Ayala s/n, Casco de Santo Tomás, Mexico City CP 11340, Mexico; 3 Laboratorio de Hemopatología, Escuela Nacional de Ciencias Biológicas, Instituto Politécnico Nacional, Carpio y Plan de Ayala s/n, Casco de Santo Tomás, Mexico City CP 11340, Mexico

**Keywords:** NK, iNKT, CD107a+, c-kit+, Lya6A+

## Abstract

The involvement of NK and other cytotoxic cells is considered the first defense line against cancer. However, a significant lack of information prevails on the possible roles played by factors considered characteristic of primitive cells, such as c-kit and Sca-1, in activating these cells, particularly in melanoma models subjected to treatments with substances under investigation, such as the case of norcantharidin. In this study, B16F1 murine melanoma cells were used to induce tumors in DBA/2 mice, estimating the proportions of NK and iNKT cells; the presence of activation (CD107a+) and primitive/activation (c-kit+/Lya6A+) markers and some tumor parameters, such as the presence of mitotic bodies, nuclear factor area, NK and iNKT cell infiltration in the tumor, infiltrated tumor area, and infiltrating lymphocyte count at 10x and 40x in specimens treated with pentoxifylline, norcantharidin, and the combination of both drugs. Possible correlations were estimated with Pearson’s correlation analysis. It should be noted that, despite having demonstrated multiple correlations, immaturity/activation markers were related to these cells’ activation. At the tumor site, iNKT cells are the ones that exert the cytotoxic potential on tumor cells, but they are confined to specific sites in the tumor. Due to the higher number of interactions of natural killer cells with tumor cells, it is concluded that the most effective treatment was PTX at 60 mg/kg + NCTD at 0.75 mg/kg.

## 1. Introduction

Using mechanisms to increase the migration and infiltration of NK and iNKT cells in different neoplasms, including melanoma, is a promising approach to treating these diseases—particularly the innate immune system cells and cytotoxic cells. Natural killer (NK) cells are an essential part of the innate immune system; they are necessary for initiating and augmenting adaptive immune responses, with a critical role in antitumor immune surveillance [1,2,3,4]. Invariant natural killer T (iNKT) cells are NK cells with invariant αβ chains that possess the innate T-cell receptor (TCR) which is able to recognize lipid antigens presented by CD1d. NK and iNKT cells exhibit potent antitumor activity through a cytotoxic response; for example, they exert direct tumor lysis, recruitment, and activation of other innate and adaptive immune cells by initiating a Th1 cytokine cascade. They also regulate immunosuppressive cells in the tumor microenvironment [5,6,7,8,9]. However, the functionality of these cells will depend on their activation and state of maturation.

For the treatment of melanoma, our team’s work demonstrated encouraging prognostic results in a murine melanoma model treated with pentoxifylline (PTX), α-galactosylceramide, and their combinations and specimens subjected to drug treatments and radiotherapy [10]. The PTX is a semi-synthetic methylxanthine derivative and has a potential role in combination therapy against cancer. Its antineoplastic effect has been described as increased susceptibility to radiation therapy and chemotherapy in different neoplasms, including melanoma. PTX protects against systemic and local side effects of chemotherapy and radiotherapy [11]. Specifically in melanoma, PTX has been tested as an adjuvant and neoadjuvant treatment at subtoxic doses and can inhibit melanoma tumor growth and angiogenesis by targeting the STAT3 signaling pathway. Its ability to inhibit the activity of the canonical WNT/β-catenin pathway in melanoma cell populations has also been demonstrated [12,13].

The way to study the immune response is varied, and one way to do it is by studying the intratumoral inflammatory infiltrate. In this context, melanoma’s tumor-infiltrating lymphocytes (TILs) are associated with a favorable prognosis and have been interpreted as an indicator of a host immune response against the tumor [14]. Similarly, the mitotic rate has been used to predict aggressive behavior in various neoplasms. In melanoma, the mitotic rate has been found to have independent prognostic value in agreement with the National Comprehensive Cancer Network criterion for indicating selective sentinel node biopsy [15]. At the same time, the nuclear factor area (NFA) can be an early indicator of cell morphological changes preceding or during apoptosis [10,16]. At the same time, the nuclear factor area (NFA) can be an early indicator of cell morphological changes preceding or during apoptosis [16]. In the melanoma model treated with PTX, a reduction in the rate of mitosis of tumor cells and an increase in leukocyte infiltrate and necroapoptosis were found [10].

Another exciting substance for the adjuvant or neoadjuvant treatment of melanoma is norcantharidin (NCTD) [17]. Its antitumor effect has been extensively investigated in different types of malignant tumors. NCTD exerts an antineoplastic effect by inhibiting tumor cell reproduction, inducing apoptosis and autophagy, restraining migration and metastasis, and affecting immunity and angiogenesis [18]. However, it has been documented that the NCTD markedly inhibits lymphoproliferation in vitro under ConA or LPS stimulation. The overhead suggests that this compound strongly suppresses lymphocyte activation in vitro [19]. NCTD inhibits melanoma growth by inducing tumor cell apoptosis by activating a TR3-dependent pathway. These results suggest that NCTD is a potential therapeutic agent for melanoma [20]. Notwithstanding the above, it is necessary to verify whether NCTD can inhibit NK and iNKT cells in a murine melanoma model and reduce their recruitment at the specific site of the tumor for their potential use as neoadjuvant or adjuvant therapy for melanoma treatment.

Despite the importance of recruitment, the degree of maturation of natural killer cells, and their activation for the control of neoplasms, there is a lack of information on whether adjuvant and neoadjuvant treatments can modify these critical parameters for melanoma control. The current study assessed the effect of PTX, NCTD, and their mixtures on NK cells and iNKT, the degree of maturation, their activation in peripheral blood, their recruitment to the tumor site, the mitotic rate, and nuclear factor area in a murine melanoma model.

## 2. Results

### 2.1. NK Cells (CD16 + CD56+), iNKT Cell (CD1D + TCRvB8.1+) in Peripheral Blood

**Figure 1:**
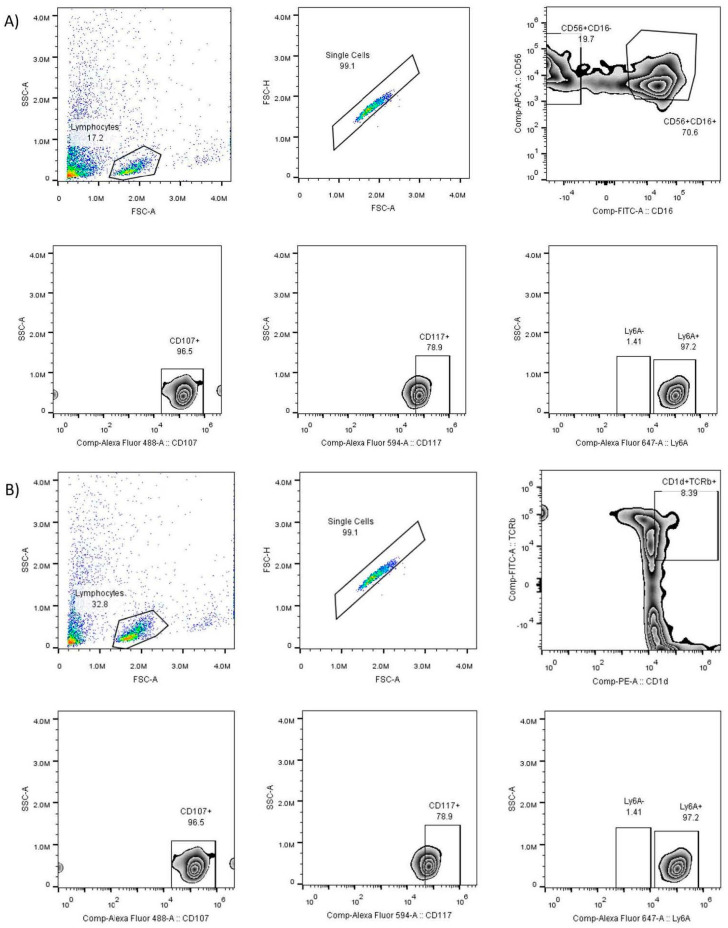
Gating strategy to identify subpopulations of natural killer cells. Panel (**A**) for NK cells shows from left to right the number of events recorded, number of lymphocytes, number of NK cells (CD56+ and CD16+ positivity), number of active NK cells (CD107+), number of NK cells with the marker c-kit (CD117+), and number of NK cells with the Sca-1 marker (Ly6A+). Panel (**B**) for iNKT cells shows, from left to right, the number of events recorded, number of lymphocytes, number of iNKT cells (CD1D+ and TCRVB8.1+ positivity), number of active iNKT cells (CD107+), number of iNKT cells with c-kit marker (CD117+), and number of iNKT cells with Sca-1 marker (Ly6A+).

Figure 1A shows the gating strategy to identify NK (CD16 + CD56+) cell populations and Figure 1B for iNKT (CD1D + TCRvB8.1+).

**Figure 2:**
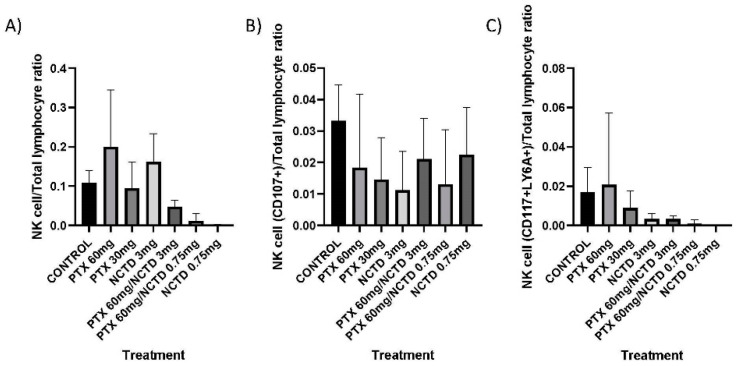
The phenotype of peripheral NK cells. (**A**) Proportion of NK cells concerning the number of total lymphocytes. (**B**) Proportion of NK cells (CD107+) to the number of total lymphocytes. (**C**) Proportion of NK cells (CD117 + LY6A +). Statistical differences to the control, *p* ≤ 0.05, *p* ≤ 0.01, and *p* ≤ 0.001.

**Figure 3:**
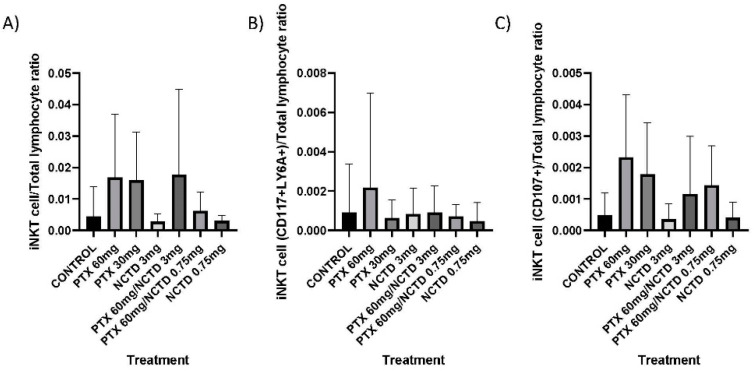
The phenotype of peripheral iNKT cells. (**A**) Proportion of iNKT cells (CD1D + TCRVB8.1+) concerning the number of total lymphocytes. (**B**) Proportion of iNKT cells (CD107+) to the number of total lymphocytes. (**C**) Proportion of iNKT cells (CD117 + LY6A +) about the number of total lymphocytes. Statistical differences concerning the control, *p* ≤ 0.05, *p* ≤ 0.01, and *p* ≤ 0.001.

The number of NK and iNKT cells in their different phenotypes in peripheral blood evaluated by flow cytometry did not present significant numerical changes in any treatment group (Figure 2A and Figure 3A, respectively). Despite this lack of differences, in the treatments with NCTD at 0.75 mg/kg, the NK cells presented the minimum value (0.011 ± 0.007 NK cell/100 lymphocytes), as was also observed in the same treatment for the iNKT (0.003 ± 0.0007 iNKT cell/100 lymphocytes) and with NCTD 3 mg/kg (0.002 ± 0.0009 iNKT cell/100 lymphocytes) with respect to their controls (NK 0.108 ± 0.010 NK cell/100 lymphocytes; iNKT 0.004 ± 0.003 iNKT cell/100 lymphocytes, respectively). This finding suggests that these cells are depleted in circulation as they are recruited into the tumor mass.

### 2.2. Activation Marker (CD107a+) on Peripheral Blood NK Cells and iNKT Cells

To assess whether norcantharidin and pentoxifylline treatment influence natural killer cell activation, we determined the ratio of CD107a+ cells to the number of total lymphocytes. All treatment groups presented fewer NK CD107a+ cells than the control group, without statistical significance (Figure 2B). However, the expression of CD107a+ in iNKT cells reveals a degranulation process. Treatment groups with PTX 60 mg/kg control, PTX 30 mg/kg, PTX 60 mg/kg in combination with NCTD 3 mg/kg, and PTX 60 mg/kg in combination with NCTD 0.75 mg/kg presented a higher proportion of iNKT CD107 cells than the control group, without statistical significance. Meanwhile, the treatment groups with NCTD 3 mg/kg and NCTD 0.75 mg/kg presented a lower proportion than the control group, without significance (Figure 3B).

### 2.3. Expression of Immaturity Markers (CD117+/LY6A+) in Peripheral Blood NK Cells and iNKT Cells

The treatment group with PTX 60 mg/kg presented a higher proportion of immature NK cells “imNK” (CD117 + LY6A +) than the control group, but there was no statistical significance. In contrast, the treatment groups with PTX 30 mg/kg, NCTD 3 mg/kg, PTX 60 mg/kg in combination with NCTD 3 mg/kg, PTX 60 mg/kg in combination with NCTD, and NCTD 0.75 mg/kg had a lower proportion of CD117 + LY6A + NK cells than the control group; however, no significant difference was calculated (Figure 2C). The treatment group with PTX 60 mg/kg presented a higher proportion of CD117 + LY6A + iNKT cells than the control group, without statistical significance. The rest of the treatment groups had a lower proportion of CD117 + LY6A + iNKT cells than the control group, without statistical significance (Figure 3C).

### 2.4. Histopathological Analysis

#### 2.4.1. Histological Findings

In the control group, the tumor cells presented large melanocytic cells (>20 microns) with poorly defined basophilic cytoplasm and a heterogeneous appearance, as well as a pleomorphic nucleus with an irregularly reinforced membrane with a heterogeneous chromatin pattern and a prominent and pleomorphic nucleolus, with a cellularity percentage of 100%. They presented extracellular pigment (melanin) with an average of 5.67 ± 0.27 mononuclear cells/10 fields—40x and tumor-infiltrating lymphocytes (TIL). In agreement with the Melanoma Institute of Australia (MIA), its grade is 1 (mild or moderate focal infiltrate, or mild multifocal infiltrate, and tumor-infiltrating lymphocytes (TILs)). According to the International Immuno-Oncology Biomarkers Working Group guidelines for evaluating TILs, the tumor presents mild TILs (5%), with an average of 4.0 ± 0.27 cells mitotic/10 fields. 

In the treatment with PTX at 60 mg/kg, the tumor was similar to that in controls; however, the average of mononuclear cells/10 fields—40x was 6.35 ± 0.21. Moreover, the tumor was Grade 1 following the MIA criteria and possessed mild TILs (5%), averaging 3.5 ± 0.21 mitotic cells/10 fields. 

The treatment with PTX as 30 mk/kg possessed an average of 6.05 ± 0.13 mononuclear cells/10 fields—40x, a finding that suggests a tumor Grade 1 following MIA criteria and mild TILs (5%), with an average of 3.8 ± 0, 26 mitotic cells/10 fields. 

In the treatment with NCTD at 3.0 mg/kg, the melanoma cells were similar to those in the control; however, the cellularity percentage was about 90%. The tumor possessed 6.13 ± 0.21 mononuclear cells/10 fields—40x and was Grade 1 according to the Ima criteria. The TILs presented an average of 3.0 ± 0.14 cells mitotic/10 fields (5%). 

The combined treatment of PTX 60 mg/kg plus NCDT at 3 mg/kg showed a cellularity percentage of 80%, with an average of 7.53 ± 0.37 mononuclear cells/10 fields—40x, following the MIA criteria. However, the evaluation of TILs suggested moderate tumor-infiltrating lymphocytes with an average of 2.1 ± 0.24 mitotic cells/10 fields.

With PTX 60 mg/kg plus NCDT at 0.75 mg/kg, the melanocytic cells were similar to in the control group, but with a cellularity percentage of 85%, with an average of 6.23 ± 0.17 mononuclear cells/10 fields—40x, following the MIA criteria. The tumor was Grade 1 = mild or moderate focal infiltrate, or mild multifocal infiltrate, tumor-infiltrating lymphocytes (TILs) with an average of 2.9 ± 0, 34 cell/10 field (Supplementary Figure S1).

#### 2.4.2. Infiltration of Mononuclear Cells in the Tumor/10 Fields—40x

**Figure 4:**
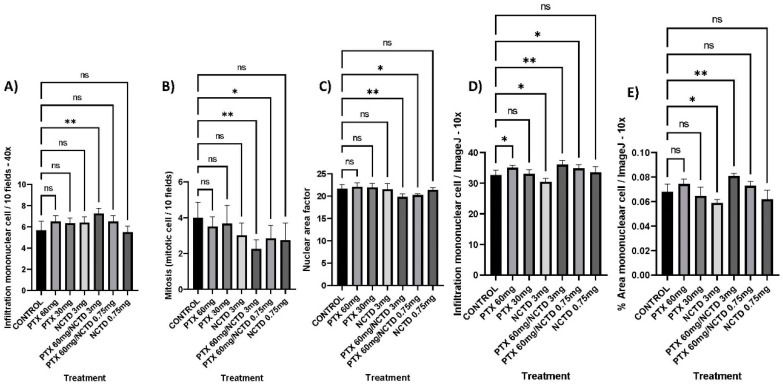
Histopathological analysis. (**A**) Infiltration of mononuclear cells/10 fields—40x. The treatment group with PTX 60 mg/kg in combination with NCTD 3 mg/kg (v.p.) showed a more significant infiltration of mononuclear cells concerning the control group, with statistical significance (** *p* ≤ 0.01). (**B**) Mitotic figures, the treatment groups with PTX 60 mg/kg in combination with NCTD 3 mg/kg and PTX 60 mg/kg in combination with NCTD 0.75 mg/kg showed a lower presence of mitotic bodies to the control group, with statistical significance (** *p* ≤ 0.01 and * *p* ≤ 0.05, respectively). (**C**) Nuclear factor area, the treatment groups with PTX 60 mg/kg in combination with NCTD 3 mg/kg and PTX 60 mg/kg in combination with NCTD 0.75 mg/kg showed a higher nuclear area factor concerning the group control, with statistical significance (** *p* ≤ 0.001 and * *p* ≤ 0.05, respectively). (**D**) Infiltration of mononuclear cell/ImageJ—10x. The treatment group with PTX 60 mg/kg in combination with NCTD 3 mg/kg (v.p.) showed a more significant infiltration of mononuclear cells concerning the control group with statistical significance (** *p* ≤ 0.01and * *p* ≤ 0.05, respectively). (**E**) % Area mononuclear cell/ImageJ—10x. The treatment group with PTX 60 mg/kg in combination with NCTD 3 mg/kg (v.p.) showed a more significant infiltration of mononuclear cells concerning the control group with statistical significance (** *p* ≤ 0.01and * *p* ≤ 0.05, respectively). ns: not significant.

The treatment group with PTX 60 mg/kg in combination with NCTD 3 mg/kg (7.25 ± 0.216 mononuclear cells/10 fields) showed a more significant infiltration of mononuclear cells in the tumor compared to the control group (5.67 ± 0.27 mononuclear cells/10 fields), with statistical significance (*p* ≤ 0.01). The treatment groups with PTX 60 mg/kg, PTX 30 mg/kg, NCTD 3 mg/kg, and PTX 60 mg/kg in combination with NCTD 0.75 mg/kg also presented with a higher amount of infiltration than the control group, but these were non-significant findings. The treatment group with NCTD 0.75 mg/kg had a lower amount of infiltration than the control group, but without statistical significance (Figure 4A).

#### 2.4.3. Mitotic Figures in the Tumoral Mass

The treatment groups with PTX 60 mg/kg in combination with NCTD 3 mg/kg (2.25 ± 0.21 mitotic cells/10 fields) and PTX 60 mg/kg in combination with NCTD 0.75 mg/kg (2.83 ± 0.28 mitotic cells/10 fields) showed a lower presence of mitotic bodies in melanoma cells compared to the control group (4.0 ± 0.27 mitotic cells/10 fields), with statistical significance (*p* ≤ 0.01 and *p* ≤ 0.05, respectively). The rest of the treatments showed a lower presence of mitotic bodies (3.66–2.75 mitotic cells/10 fields) concerning the control group, but without statistical significance (Figure 4B).

#### 2.4.4. Nuclear Factor Area (NFA) of Melanoma Cells

The treatment groups with PTX 60 mg/kg in combination with NCTD 3 mg/kg (19.8 ± 0.3 NFA) and PTX 60 mg/kg in combination with NCTD 0.75 mg/kg (20.21 ± 0.13 NFA) showed less nuclear area factor more significant than the control group (21 ± 0.35 NFA) with statistical significance (*p* ≤ 0.001 and *p* ≤ 0.05, respectively). The rest of the treatments did not show statistical significance (Figure 4C).

#### 2.4.5. Infiltration of Mononuclear Cells in the Tumor/ImageJ—10x

The treatment group with PTX 60 mg/kg combined with NCTD 3 mg/kg showed a more significant infiltration of mononuclear cells in the tumor than the control group, with statistical significance (*p* ≤ 0.01). The treatment groups with PTX 60 mg/kg and PTX 60 mg/kg in combination with NCTD 0.75 mg/kg also presented higher infiltration than the control group, with statistical significance (*p* ≤ 0.05). The treatment group with NCTD 3 mg/kg had a lower infiltration than the control group, with statistical significance (*p* ≤ 0.05) (Figure 4D).

#### 2.4.6. Tumoral Area Infiltrated with Mononuclear Cell/ImageJ—10x

The treatment group with PTX 60 mg/kg combined with NCTD 3 mg/kg showed a more significant infiltration of the area with mononuclear cells in the tumor than the control group with statistical significance (*p* ≤ 0.01). The treatment group with NCTD 3 mg/kg had a lower infiltration of the area with mononuclear cells than the control group with statistical significance (*p* ≤ 0.05) (Figure 4E).

### 2.5. Detection of NK and iNKT in the Tumor Mass by Immunofluorescence Microscopy

**Figure 5:**
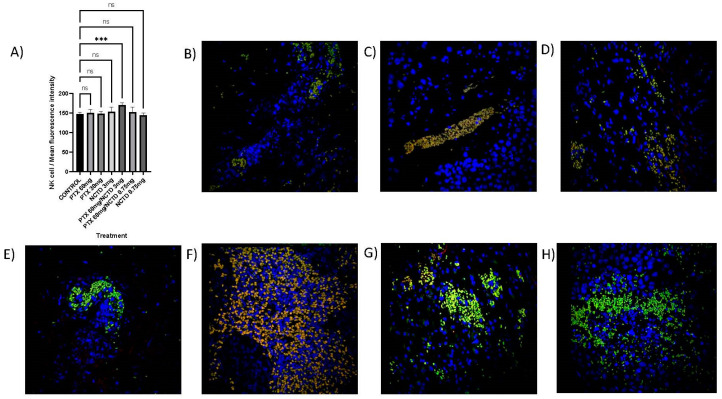
Immunofluorescence microscopy image of merged NK cells (CD16+/CD56+/DAPI+) by treatment group. (**A**) Immunofluorescence microscopy analysis, of intratumoral lymphocytic infiltration by NK cells (CD16 + CD56+) with statistical significance for the treatment group with PTX 60 mg/kg in combination with NCTD 3 mg/kg (*** *p* ≤ 0.001). No statistically significant difference was detected in the rest of the treatments. (**B**) Control group, sterile saline solution. (**C**) PTX 60 mg/kg. (**D**) PTX 30 mg/kg. (**E**) NCTD 3 mg/kg. (**F**) PTX 60 mg/kg + NCTD 3 mg/kg. (**G**) PTX 60 mg + NCTD 0.75 mg/kg. (**H**) NCTD 0.75 mg/kg. ns: not significant.

**Figure 6:**
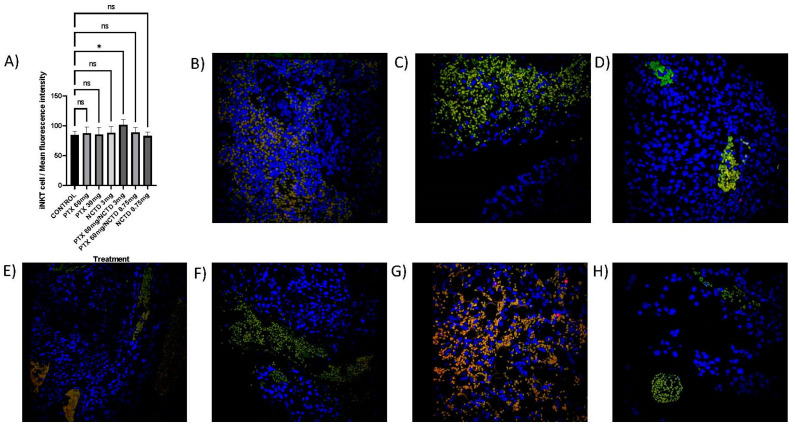
Immunofluorescence microscopy image of iNKT cells in merged (CD1D+/TCRVB8.1+/DAPI+) by treatment group. (**A**) Immunofluorescence microscopy analysis of intratumoral lymphocytic infiltration by iNKT cells (CD1D + TCRVB8.1+) with statistical significance for the treatment group with PTX 60 mg/kg in combination with NCTD 3 mg/kg, (* *p* < 0.05). (**B**) Control group, sterile saline solution. (**C**) PTX 60 mg/kg. (**D**) PTX 30 mg/kg. (**E**) NCTD 3 mg/kg. (**F**) PTX 60 mg/kg + NCTD 3 mg/kg. (**G**) PTX 60 mg + NCTD 0.75 mg/kg. (**H**) NCTD 0.75 mg/kg. ns: not significant.

The immunofluorescence analysis confirmed intratumoral lymphocytic infiltration of NK cells (Figure 5A–H) and iNKT (Figure 6A–H). Interestingly, in the treatment of PTX 60 mg/kg in combination with NCTD 3 mg/kg, a significant increase (*p* ≤ 0.001) of NK cells (169.75 ± 2.63 mean intensity fluorescence) was found concerning the control group (146.55 ± 1.64 mean intensity fluorescence) (Figure 5A,G) and also for iNKT cells (101.25 ± 4.12 mean intensity fluorescence; *p* ≤ 0.05) compared to the control group (82.75 ± 2.77 mean intensity fluorescence) (Figure 6A,G). No statistically significant difference was detected in the rest of the treatments.

### 2.6. Relationships between Natural Killer Cells with Their Recruitment to the Tumor Site and Their Cytotoxic Effects on Melanoma Cells

Significant relationships were found through the Pearson correlation analysis of tumor-site infiltrated or peripheral blood natural killer cells with some tumor cell variables such as apoptosis and nuclear area factor and other response data immune at the tumor site as the lymphocytic infiltrate in the tumor at 40x; the lymphocyte count at 10X and the infiltrated tumor area in the murine model of melanoma treated with PTX and NCTD and their mixtures are shown in Table 1. It should be noted that immaturity/activation markers (c+kit+/Lya6A+) were related to these cells’ activation (CD107a+). At the tumor site, iNKT cells are the ones that exert the cytotoxic potential on tumor cells, but they are confined to specific sites in the tumor.

## 3. Discussion

The innate ability to detect tumor cells of NK and iNKT cells makes them critical in antitumor immune surveillance [21,22] through the release of a potent cytotoxic response, which is why they have become, per se, a therapeutic target against melanoma [23]. Our study is one of the first to evaluate the effects of pentoxifylline (PTX) and norcantharidin (NCTD) as a therapeutic target against melanoma by evaluating the presence, activation, and degree of maturity of NK and iNKT cells in peripheral blood and their infiltration in the tumor. Our work group previously demonstrated that PTX increased the leukocyte infiltration into the tumor mass (line B16-F1 melanoma cell) related to a good prognosis [10]. Similarly, NCTD treatment can decrease the number of tumor-infiltrating Tregs and increase the number of CD4+ and CD8+ T cells in prostate cancer cells [24]. Considering these findings and the background described above, it was hypothesized that PTX and NCTD influence the number and recruitment of NK and iNKT cells in a mouse melanoma model.

The number of NK and iNKT cells in their different phenotypes in peripheral blood evaluated by flow cytometry did not present significant numerical changes in any treatment group. This finding suggests that these cells are depleted in circulation, as they are recruited into the tumor mass. However, the Pearson correlation analysis showed that, in the murine model of melanoma, in addition to the recruitment of natural killer cells to the tumor site, a series of relatively complex events occur, both in specimens without drug treatment and those subjected to different drug schemes with PTX, NCTD, and their mixtures, which also depend on the test doses.

In specimens with melanoma tumors, NK cells (CD16 + CD56+) and iNKT cells (CD1D + TCRvB8.1) from peripheral blood are active (CD107a+) and preserve immature markers, such as Sca-1+, in addition to the transcription factor/immaturity c-kit (CD117+). The lysosome-associated membrane glycoprotein 1 (LAMP-1 or CD107a) has been described as a marker of the functional activity of NK cells associated with the secretion of lytic granules containing molecules such as perforin and granzymes for combating the disease [25,26,27,28]. An exciting finding in the mouse melanoma model is that the activated natural killer cells require or are c-kit+/Sca-1+, suggesting that these markers of immaturity are involved in natural killer cell proliferation, survival, and activity. T-cell acute lymphocytic leukemia protein 1 “SCF” or “SCL,” also known as c-kit ligand, promotes the growth of lineage-specific hematopoietic precursor cells. In addition, this protein synergizes with hematopoietic growth factors such as erythropoietin, granulocyte colony-stimulating factor (G-CSF), and granulocyte/macrophage–colony-stimulating factor (GM-CSF) [29]. The helix–loop–helix heterodimer (SCL, most probably coupled with bHLH E12/E47) DNA-binding domain acts as a transcription factor [28,30]. In the specific case of NK cells in a mouse-melanoma model, this binding promotes the survival of peripheral CD16 + CD56+c-kit+Sca-1+ cells. The absence of c-kit signaling has also been shown to reduce the generation of NK cells from fetal liver precursors [31]. Although stem-cell antigen 1 (Sca-1, also named Ly-6A/E) has been best characterized as a marker of hematopoietic stem cells. In the context of NK cells, Sca-1 is a marker of early nonselective NK cell activation associated with increased IFN-γ production, thereby promoting apoptosis and cytolysis of target tumor cells [32,33]. Furthermore, the expression of Sca-1 in NK cells may be involved in the terminal differentiation of NK cells under the stimulus of IL-12. As a result, high cytotoxicity and high potential to produce IFN-γ are present in pre-NK cells [28], that is, those that preserve primitiveness markers.

Moreover, in the melanoma model without drug treatment, it was observed that the leukocyte infiltrates in the tumor area assessed at 10x were inversely related to peripheral blood NK cells. Tumor-infiltrating lymphocytes such as CD+8 in patients with melanoma are inversely related to sentinel lymph node positivity and directly correlated with a statistically significant improvement in both melanoma-specific survival time and with the recurrence-free of disease [24].

Enigmatically, tumor cells’ nuclear factor area (NFA) was inversely related to peripheral blood NK cells expressing immaturity factors such as Sca-1 and c-kit in the melanoma control group. NFA can be an early indicator of cell morphological changes that occur during apoptosis, and a quantitative measure of apoptosis can be obtained through image analysis [16]. Although these immaturity markers give NK cells a higher cytotoxic capacity [28,32,33], probably the growth of tumor cells interferes with the lack of maturation of NK cells. In addition, it has been shown that, in multiple neoplasms, including breast cancer, colon cancer, and melanoma cell lines, the growth of these malignant cells can interrupt the functional maturation of NK cells and impair the antitumor capacity of NK cells [34]. The previous report and the findings of this study suggest that B16-F1 cells impede the maturation of NK cells at the bone-marrow level as a side effect of their growth in animals with melanoma without drug treatment. In addition, NK cells are not significantly recruited to the tumor site under this condition. There are no previous results on this particular subject; however, from the murine melanoma model without drug treatment, it was possible to demonstrate the relevance of transcription factors such as c-kit/Sca-1 for activating peripheral blood NK cells and iNKT.

In the specimens treated with PTX at 60 mg/kg, active peripheral blood NK cells that expressed c-kit+Sca-1+ were present. This result reinforces the hypothesis that these immaturity markers favor its activation through LAMP-1, also probably regulated by c-kit. Likewise, high doses of PTX can “orient” the response of natural killer cells so that both cell subpopulations remain active and correlated. Interestingly, and as documented in mice without drug treatment, immaturity markers (c-kit+/Sca-1+) are required for iNKT activation and correlated with active NK in peripheral blood in this treatment. However, it was found that the number of mitotic bodies in the tumor cells was directly related to the number of NK cells in peripheral blood. In patients, tumor mitotic body count is a significant predictor, potentially improving melanoma staging accuracy and defining their risk categories [14,35,36]. 

In the same way, in the treatment with PTX at 30 mg/kg, the tumor mitotic body count in the melanoma cells presented a direct relationship with the lymphocytic infiltrate. The statistical associations seem paradoxical; however, it is possible to emphasize the following. Tumor-infiltrating lymphocytes (TILs) comprise effector T lymphocytes, regulatory T lymphocytes, NK cells, dendritic cells, and macrophages. Nevertheless, its distribution and activation status may be variable and modulate clinical outcomes [37]. In addition, melanoma tumor cells can employ different immune evasion strategies, such as downregulating MHC class I molecules to avoid recognizing CD8+ T cells [38]. Likewise, melanoma cells interfere with some interleukins’ secretion and activate inhibitory immune checkpoints [39,40]. The mechanism of inhibition elicited by melanoma cells on NK cells probably consists of TGF-β release, an increase in MHC I expression, and limiting the availability of IL-2 performed by Tregs [39]. The findings of this study suggest that, under the treatments with PTX alone (60 and 30 mg/kg), other cell populations with cytotoxic capacity participate against the tumor, in addition to the effect of the drug itself [10]. 

Peripheral blood iNKT cells from mice given the low dose of NCTD (0.75 mg/kg) were active. They displayed markers of immaturity/activation, even though, in the higher dose of NCTD (3.0 mg/kg), the activation of NK cells was also related to these markers of immaturity, as observed in the control group. This statistical relationship again corroborates the importance of c-kit as a transcription factor involved in hematopoietic development and increased cytotoxic potential [28,30,40,41,42,43,44]. These results contrast those found in vitro, where NCTD was shown to suppress lymphocyte activation [19], which is very likely to occur in vitro since NCTD is a potent inhibitor of protein phosphatases 1, 2A, 2B, and 5 [45].

Interestingly, in mice treated with NCTD at 3.0 mg/k, the lymphocyte count in the tumor mass was closely related to peripheral blood iNKT/CD107a+ cells expressing Sca-1 + CD117+. The migration of NK cells to the tumors depends, among other things, on the expression of the CXCR3 ligand and CXCL10 [46]. B16-F10 cells with a low expression of CCL5 display chemotactic activity and induce the migration of various subsets of immune cells to inflammatory sites through three chemokine receptors: CCR1, CCR3, and CCR5 [46]. In patients, higher CCL5 levels correlate with significantly longer survival [47]. Another factor that could influence this is the multifunctional protein acting through the receptor ChemR23/CMKLR1, which is present in B16 cells. This biomolecule has antiangiogenic properties that are also linked to the induction of necrosis, and it mediates the migration and recruitment of plasmacytoid dendritic cells and ChemR23-dependent NK cells [48,49]. Therefore, treatment with NCTD at a dose of 3.0 mg/kg, in addition to inducing a cytotoxic pharmacological effect on tumor cells [17], may favor the migration of iNKT cells to the tumor site. Moreover, a significant part of the tumor-infiltrating lymphocytes is active (CD107a+) and presented immature markers under this treatment. 

Tumor-infiltrated iNKT cells were inversely related to tumor-infiltrated areas. This finding proposes the presence of chemotactic factors that allow their accumulation at specific tumor sites. About the topic, chemokines are closely associated with iNKT cell maturation and localization, specifically CCL3 and CCL4, accompanied by their CXCR3 ligands (CXCL9-11) secreted by activated dendritic cells. These chemokines attract different iNKT subsets to chemokine/ligand sites [50,51]. Furthermore, interleukins activate iNKT cells by binding to receptors on the cell surface [52]. Specifically, IL-27 modulates IL-12 secretion from dendritic cells, indirectly enhancing the maintenance and recruitment of iNKT cells [53,54]. Furthermore, some lipid antigens presented by dendritic cells to iNKT cells through CD1d allow their proliferation at specific sites [10,55,56,57]. Additionally, in the treatment with NCTD at 0.75 mg/kg, the total area infiltrated by lymphocytes was negatively related to the mitosis rate of the tumor cells. This discovery suggests the role of CD8+ T cells and iNKT cells in the depletion of tumor cells, in addition to the effect of the drugs themselves.

The current study tested, for the first time, whether the effect of PTX in combination with NCTD in a mouse melanoma model can induce cell death, decrease the rate of mitosis, and improve the antitumor immunity by recruiting NK cells and iNKT at the tumor site. Through Pearson’s correlation analysis, we saw that NK cells infiltrated the tumor and reached a significant maximum in the treatment of PTX at 60 mg/kg + NCTD at 3.0 mg/kg, and iNKT cells in the tumor were negatively correlated with peripheral blood NK cells. These findings corroborate the migration of these peripheral blood natural killer cells to the tumor site, as has been reported for other types of cancer, including melanoma [58,59,60]. The histopathological analysis revealed that NCTD, in combination with PTX in both treatments, increased the number of infiltrating lymphocytic cells in the tumor mass. With PTX 60 mg/kg + NCTD 3.0 mg/kg, iNKT cells at the tumor site were inversely related to NK cells from peripheral blood. Infiltration of cytotoxic NK cells and iNKT cells into tumors is a favorable prognostic marker for various cancers, including melanoma [23].

Furthermore, NK cells isolated from an active site of inflammation possess enhanced functional capabilities [58,59]. In patients with melanoma, tumor-infiltrating lymphocytes confer more remarkable survival and prolong disease-free lifetime [24]. The recruitment and functioning of NK and iNKT cells on the tumor by administering NCTD and PTX were corroborated by the mitotic index and the nuclear area factor (sensitive predictor of the early apoptotic effect of anticancer therapy). In addition, there was a lower presence of mitotic bodies in B16-F1 cells compared to the control group. Consequently, the highly significant inverse correlations (r^2^ = −1) between the mitotic bodies and the infiltrated lymphocyte count (40x) in the treatment of PTX at 60 mg/kg + NCTD at 3.0 mg/kg were found. This result demonstrates that cytotoxic cells participate in the fate of the tumor the greater the number of infiltrated lymphocytes and the lower the replication rate of the tumor cells.

In the same sense, in the treatment of PTX at 60 mg/kg + NCTD at 0.75 mg/kg, a significant correlation was observed between iNKT cells expressing Sca-1+/c-kit+ with the lymphocytic infiltrate (40x), and the NFA had a direct correlation with peripheral blood iNKT. Similarly, in the PTX 60 mg/kg + NCTD 3.0 mg/kg, the NFA of the tumor cells was increased concerning the control; however, it did not correlate with the NK or iNKT cells of peripheral blood. These correlations suggest that combining both drugs elicits this damage preceding tumor cell apoptosis. Furthermore, PTX has been shown to induce apoptosis in A375 and MeWo human melanoma cell lines [60,61] and in squamous cell carcinoma [62], and it has also been found in a mouse-melanoma model induced with B16-F1 cells [10]. Meanwhile, NCTD exerts this same damage in human melanoma A375 cells [63,64] and in WM35 and 1205Lu melanoma cell lines. Moreover, it can also be found in a transgenic mouse model expressing BRAFV600E [20]. Due to the characteristic changes in nuclear morphology during apoptosis, morphological features can be used as indicators of the activation of programmed cell death. A low NFA has been reported as an early sign of cell death [65,66,67,68]. However, the participation of peripheral-blood iNKTs in NFA induction is ruled out since the induction of cytotoxic damage induced by these cells occurs by direct contact with the target cell.

## 4. Materials and Methods

### 4.1. Tumor Induction

DBA/2 mice (6–9 weeks old) weighing 30 g, provided by the Environmental Toxicology Laboratory of the National School of Biological Sciences of the National Polytechnic Institute, were used. Mice were maintained in the laboratory in 12-hour cycles: 12 h light/dark at 23 °C and 40–60% humidity with water and food ad libitum. Solid tumor induction was performed by subcutaneously administering 0.7 × 10^6^ murine melanoma B16-F1 cells (ATCC^®^ CRL-6323^TM^ lot 64346031) on the back of mice. Random groups were formed (6 mice per group) once the tumor was measurable (±5.0 mm). The specimens were managed in agreement with Article 38 and Chapter V of the Directive 2010/63/EU of the European Parliament and of the Council of 22 September 2010 on the protection of animals used for scientific purposes (https://eur-lex.europa.eu/legal-content/EN/TXT/?uri=celex%3A32010L0063, accessed on 6 October 2023). The current study was reviewed and approved by the ENCB Bioethics Committee, with license number CEI-ENCB-ZOO-021-2020.

### 4.2. Pharmacological Treatment

**Figure 7:**
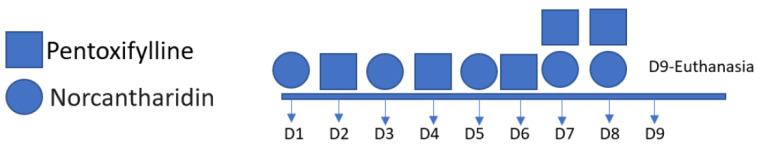
The figure shows the days of drug administration: pentoxifylline was administered on days 2, 4, 6, 7, and 8; norcantharidin was administered on days 1, 3, 5, 7, and 8.

Norcantharidin (NCTD, CAS number 5442-12-6) Sigma^®^, Livonia, MI, USA; pentoxifylline (PTX, CAS number 6493-05-6) Sigma^®^, USA, dissolved in sterile saline solution; and their combination were administered intraperitoneally (i.p.), forming seven groups, as follows: Group 1—control, sterile saline solution; Group 2—PTX 60 mg/kg; Group 3—PTX 30 mg/kg; Group 4—NCTD 3 mg/kg; Group 5—PTX 60 mg/kg + NCTD 3 mg/kg; Group 6—PTX 60 mg + NCTD 0.75 mg/kg; and Group 7—NCTD 0.75 mg/kg. The treatment was administered by being interspersed in five doses, as shown in Figure 7. The test doses were established based on the previous results from our work group for PTX [10], and the doses considered safe for NCTD without systemic toxic effects were selected based on the study by Martínez-Razo et al. [21].

### 4.3. Sample Processing

At the end of the treatment administration, the mice were anesthetized with isoflurane, and a blood sample was extracted from each specimen by cardiac puncture and collected in tubes with EDTA anticoagulant. Subsequently, the mice were euthanized with excess sodium pentobarbital and cervical dislocation to obtain the tumor fixed in 10% buffered formaldehyde, pH 7.0.

### 4.4. Flow Cytometry

Peripheral blood mononuclear cells (PBMCs) were isolated by employing a density-gradient method, using Ficoll-Paque^TM^ PLUS (GE Healthcare, Life Sciences, Lancaster, PA, USA). Freshly isolated PBMCs were fixed and permeabilized with a Fixation and Cell Permeabilization Kit (Invitrogen, Waltham, MA, USA) and labeled with appropriate dilutions of the following specific antibodies labeled with different fluorochromes at a 1:3000 dilution. For iNKT, PE anti-mouse CD1d (K253, BioLegend, San Diego, CA, USA) and FITC anti-mouse TCRvB8.1 (KJ16-133.18, BioLegend, USA) were used. For NK, APC anti-human CD56 (HCD56, BioLegend, USA) and FITC anti-mouse CD16 (93, BioLegend, USA) were used. As an activation biomarker, AlexaFluor^®^488 anti-mouse CD107 (H4A3, BioLegend, USA) was used. Primitiveness biomarkers were AlexaFluor^®^647 anti-mouse LY6A (1755, Southern Biotech, USA) and unconjugated anti-mouse CD117/c-Kit (Poly16832, Abcam, USA) that was detected using a secondary Ab labeled with AlexaFluor^®^594 (AF 594 anti-mouse IgG, Poly4053, BioLegend, USA). After staining, samples were washed with Cell Staining Buffer (BioLegend, 420201, USA) and resuspended in 4% paraformaldehyde for immediate flow cytometric evaluation.

A Cytek Northern Lights™ cytometer (Biosciences, State College, PA, USA) from the Flow Cytometry-Instrument Center of the National Medical Center, Siglo XXI, IMSS was used by employing the SpectroFlo software (https://cytekbio.com/pages/spectro-flo). Microbead tubes (Anti-Mouse Ig, κ/Negative Control Compensation Particles Set, BD^TM^ CompBead, BD Biosciences, USA) were stained with individual fluorochrome-labeled antibodies and served to establish a compensation matrix. Cells were gated based on their forward/side scatter characteristics and fluorescence minus one (FMO) control for each marker. NK cells were defined as CD16 + CD56+, and iNKT cells were defined as CD1D + TCRvB8.1+. Immature NK and iNKT cells were defined as CD16 + CD56 + LY6A + CD117+ and CD1D + TCRvB8.1 + LY6A + CD117+, respectively. The activation of NK and INKT cells was defined as CD16 + CD56 + CD107a+ and CD1D + TCRvB8.1 + CD107a+, respectively. Set compensation and frequency calculations of specific cellular subsets were performed using FlowJo (FlowJo, LLC, Ashland, OR, USA).

### 4.5. Histopathological Analysis

Tumor tissues were processed with the paraffin-embedding technique, obtaining tissue sections 4 μm thick. One of the sections was stained with hematoxylin–eosin (H&E). The histological description of the tumor, the mononuclear cell count, and mitotic figures were made from the taking of 10 images per specimen of each treatment where the solid neoplasm was located without necrosis or hemorrhage through ImageJ Version 1.53t (National Institutes of Health, Bethesda, MD, USA; available from https://imagej.nih.gov/ij/).

### 4.6. Analysis of Cell Nuclear Morphology (Nuclear Factor Area) and Distribution in the Tumor Mass

The ImageJ software, developed by the National Institutes of Health (NIH), is a valuable tool for evaluating and discriminating apoptotic cells, where the nuclear factor area (NAF) can be an early indicator of the cellular morphological changes that occur during apoptosis. Thus, NFA can be used to obtain a quantitative measure of apoptosis [17]. The evaluation of the images (40x) was carried out using ImageJ Version 1.53t of the National Institutes of Health, Bethesda, available at https://imagej.nih.gov/ij/. Photomicrographs of tissues stained with hematoxylin and eosin were converted to 8-bit images and then auto-thresholded using the default method by the “Make Binary” function. After that, the “Analyze Particles” function was used to analyze nuclear morphology. The surface area, the perimeter (the length of the selection’s outer limit), and the nuclei’s circularity were automatically measured. Then, the nuclear factor area (NFA) was calculated by the product of the nuclear area and the circularity.

Mononuclear cell-infiltrated area (10x) in the tumor: Image evaluation was performed using ImageJ Version 1.53t. H&E-stained photomicrographs were converted to 8-bit images and then auto-thresholded by the “Make Binary” function, using the default method. After that, the “Analyze Particles” function was used to determine the number of tumor-infiltrating cells and the area they occupied concerning the tumor mass.

### 4.7. Detection of NK and iNKT Cells by Immunofluorescence in the Tumor Mass

Another section of the fixed tissue was subjected to immunohistochemical detection of NK and iNKT cells, using fluorochrome-labeled specific antibodies diluted 1:1000: PE anti-mouse CD1d (K253, BioLegend, USA), FITC anti-mouse TCRvB8.1 (KJ16-133.18, BioLegend, USA), APC anti-human CD56 (HCD56, BioLegend, USA), and FITC anti-mouse CD16 (93, BioLegend, USA). Nuclei were stained with DAPI (Molecular Probes^TM^). NK cells were defined as CD16 + CD56+, iNKT cells were defined as CD1D + TCRvB8.1+, samples were analyzed using a confocal laser scanning microscope (Olympus IX71 inverted microscope), and the image analysis (mean fluorescence intensity) was performed using ImageJ Version 1.53t.

### 4.8. Statistical Analysis

The results were analyzed by ANOVA, followed by the post hoc Dunnett test. All *p*-values ≤ 0.05 were considered statistically significant. GraphPad Prism version 7.0 software (GraphPad Software, San Diego, CA, USA) was used for statistical analysis and graphic representation. The results were logarithmically transformed and subjected to a Pearson correlation analysis, using the GraphPad software to analyze the individual relationship between the peripheral blood variables obtained by flow cytometry and the morphological variables of histopathological and immunohistochemical analysis. Results with a *p* ≤ 0.05 were considered significant.

## 5. Conclusions

**Figure 8:**
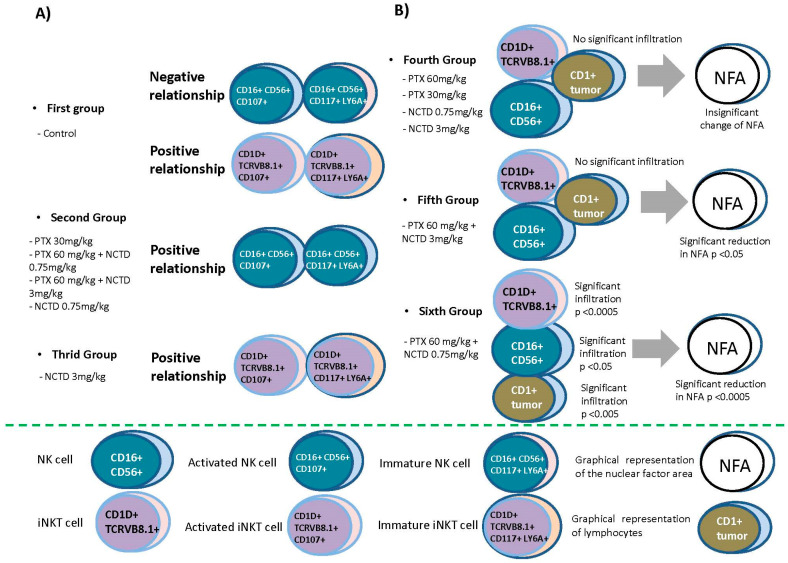
Graphic representation of the correlations of the natural killer cell subpopulations (NK and iNKT) in peripheral blood and at the tumor site in a mouse melanoma model treated with pentoxifylline (PTX), norcantharidin (NCTD), and their mixtures. (**A**) Peripheral blood. According to the evaluation by flow cytometry, the following distribution groups of the subpopulations of NK and iNKT cells were formed. (**B**) Melanoma tumor infiltration of NK and iNKT cells by Confocal microscope.

PTX, NCTD, and their mixtures can improve the treatment of melanoma in a mouse model, mainly using the combination of PTX at 60 mg/kg + NCTD at 0.75 mg/kg. This statement is feasible since significant correlations of iNKT cells with lymphocyte infiltration and with infiltrated tumor areas were observed. iNKT cells are actively presenting CD107a+; however, this association is regulated by the expression of T-cell acute lymphocytic leukemia protein 1 “SCF” or “SCL,” also known as c-kit ligand, which participates as a transcription factor in DNA, and by the stem cell antigen 1 (Sca-1), which probably increases the cytotoxic capacity of iNKTs through the secretion of perforins and granzymes due to the positivity of the lysosome-associated membrane glycoprotein 1 (LAMP-1). Thus, the treatments with PTX, NCTD, and their mixtures exert their antitumor activity through the effect of the drugs themselves and by the recruitment of iNKT cells to the tumor site. However, the correlations between the variables under study are complex in the different treatments (Figure 8), highlighting the active participation of the iNKT infiltrating the tumor site.

## Figures and Tables

**Table 1:** Pearson correlation analysis shows significant correlations of natural killer NK (CD16 + CD56+) and iNKT (CD1D + TCRvB8.1+) cells, either in an activated state (CD107a+) or presenting immaturity/activation markers (c-kit+/Lya6A+) in a mouse model of melanoma. The relationships of these cells in peripheral blood or infiltrates at the tumor site concerning some variables analyzed in the tumor are presented.

Treatment	Correlated Variables		r^2^	*p*-Value
Control	NKc-kit/Lya6A	NK107a	−0.668	*p* ≤ 0.05
	iNKT	iNKT107a	0.890	*p* ≤ 0.01
	iNKT	iNKT/c-kit/LyA6A	0.982	*p* ≤ 0.001
	iNKTc-kit/LyA6A	iNKT107a	0.796	*p* ≤ 0.05
	NKc-kit/Lya6A	NFA	−0.704	*p* ≤ 0.05
PTX 60 mg/kg	NKc-kit/LyA6A	NK107a	0.957	*p* ≤ 0.01
	NK107a	iNKT107a	0.929	*p* ≤ 0.01
	NK107a	iNKTc-kit/Lya6A	0.885	*p* ≤ 0.05
	NKc-kit/Lya6A	iNKT107a	0.849	*p* ≤ 0.05
	NKc-kit/Lya6A	iNKTc-kit/Lya6A	0.977	*p* ≤ 0.001
	NK	Mitosis	0.895	*p* ≤ 0.05
PTX 30 mg/kg	NKc-kit/Lya6A	NK107a	0.878	*p* ≤ 0.05
	L.I. 40x	Mitosis	1.0	*p* ≤ 0.001
NCTD 3.0 mg/kg	iNKTc-kit/Lya6A	iNKT107a	0.970	*p* ≤ 0.01
	iNKT107a	L.C. 10x	0.875	*p* ≤ 0.05
	iNKTc-kit/LyA6A	L.C. 10x	0.870	*p* ≤ 0.05
	iNKT tumor	Area	−0.983	*p* ≤ 0.01
NCTD 0.75 mg/kg	NK	NK107a	0.987	*p* ≤ 0.05
	NKc-kit/Lya6A	NK107a	0.967	*p* ≤ 0.05
	NK tumor	Mitosis	0.972	*p* ≤ 0.05
	NK	iNKT tumor	0.959	*p* ≤ 0.05
	NK107a	iNKT tumor	0.943	*p* ≤ 0.05
	Mitosis	Area	−0.993	*p* ≤ 0.01
	NK tumor	Area	−0.992	*p* ≤ 0.01
PTX 60 mg/kg + NCTD 3.0 mg/kg	NKc-kit/Lya6A	NK107a	0.991	*p* ≤ 0.01
	Mitosis	L.I. 40x	−1.0	*p* ≤ 0.001
	NK	iNKT tumor	−0.949	*p* ≤ 0.05
	iNKT107a	L.C. 10x	0.944	*p* ≤ 0.05
PTX 60mg/kg + NCTD 0.75 mg/kg	NK	NK107a	0.97	*p* ≤ 0.001
	NK	NKc-kit/Lya6A	0.994	*p* ≤ 0.001
	NKc-kit/Lya6A	NK107a	0.945	*p* ≤ 0.01
	iNKTc-kit/Lya6A	L.I. 40x	0.941	*p* ≤ 0.01
	iNKT	NFA	0.886	*p* ≤ 0.05
	iNKT	NK tumor	0.828	*p* ≤ 0.05
	NK tumor	L.I. 40x	−0.857	*p* ≤ 0.05
	iNKTc-kit/Lya6A	iNKT tumor	0.837	*p* ≤ 0.05
	L.C. 10x	Area	0.874	*p* ≤ 0.05

L.I., lymphocytic infiltrate in tumor; L.C., lymphocyte count; Area, infiltrated tumor area.

## Data Availability

Data is contained within the article.

## Supplementary Materials

The following supporting information can be downloaded at: https://www.mdpi.com/article/10.3390/ph16101472/s1. Figure S1: Hematoxylin-eosin (H&E) staining, histological description of the tumor—40x: (A): CONTROL, sterile saline solution. (B) PTX 60 mg/kg. (C) PTX 30 mg/kg. (D) NCTD 3 mg/kg. (E) PTX 60 mg/kg + NCTD 3 mg/kg. (F) PTX 60 mg + NCTD 0.75mg/kg. Figure S2. Cell staining of NK cells in melanoma sections. (A) Without fluorescence. (B) DAPI-stained nuclei. (C) CD16/32. (D) CD56. (E) Merged. Figure S3. Localization of iNKT cells in melanoma sections. (A) No fluorescence. (B) DAPI-stained nuclei. (C) TCRVB8.1. (D) CD1D. (E) Merged.
