# Supplementary material for: Tumor-Infiltrating iNKT Cells Activated through c-Kit/Sca-1 Are Induced by Pentoxifylline, Norcantharidin, and Their Mixtures for Killing Murine Melanoma Cells
Source: Pharmaceuticals (Basel). 2023 Oct 16;16(10):1472. doi: 10.3390/ph16101472 (PMC10610189; doi:10.3390/ph16101472)
Supplement: Supplementary file 1 [file pharmaceuticals-16-01472-s001.zip › pharmaceuticals-2623432-supplementary.pdf]

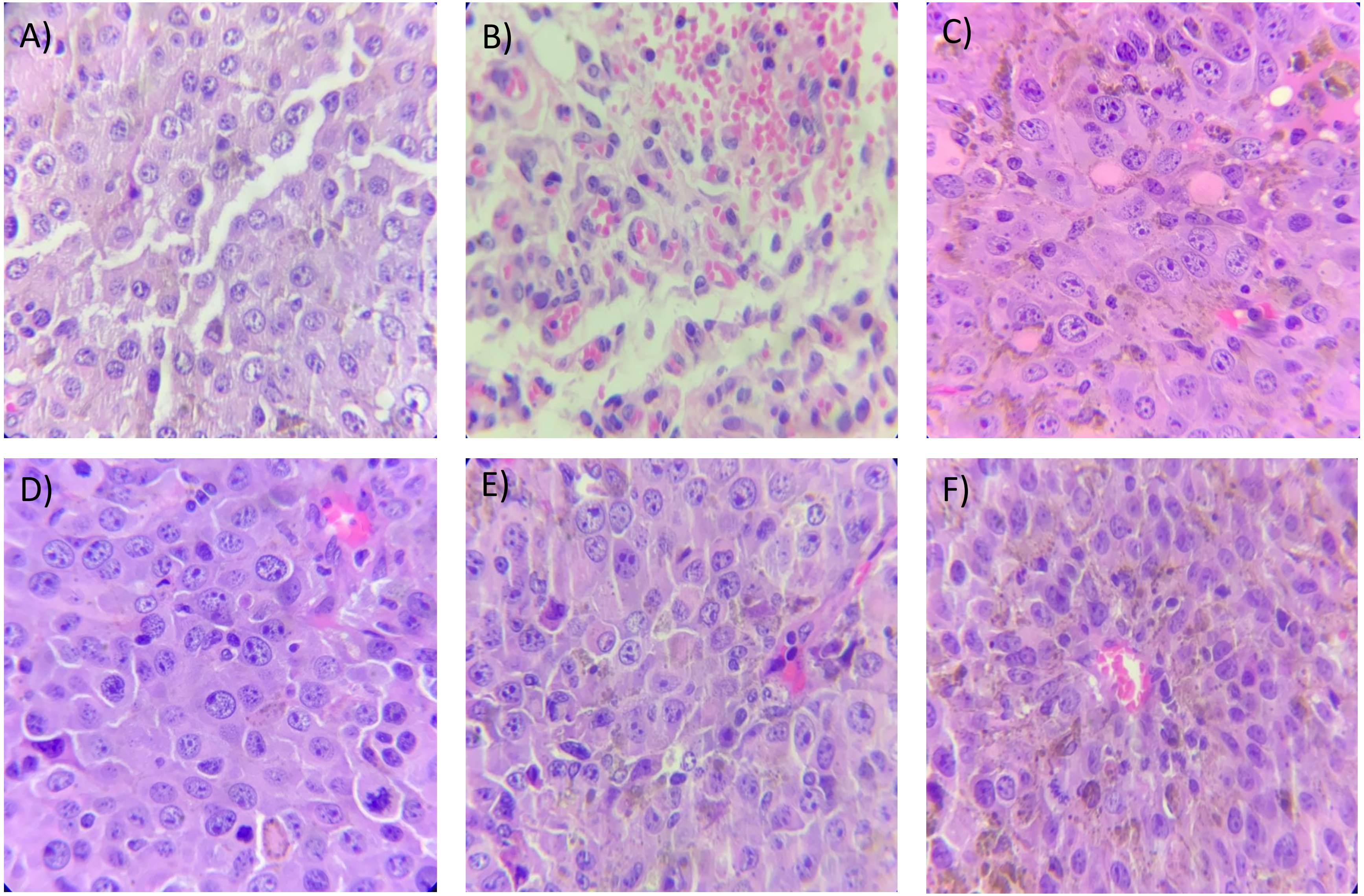

Supplementary Figure S1: Hematoxylin-eosin (H&E) staining, histological description of the tumor – 40x: A): CONTROL, sterile saline solution. B) PTX 60 mg/kg. C) PTX 30 mg/kg. D) NCTD 3mg/kg. E) PTX 60 mg/kg + NCTD 3 mg/kg. F) PTX 60mg + NCTD 0.75mg/kg.

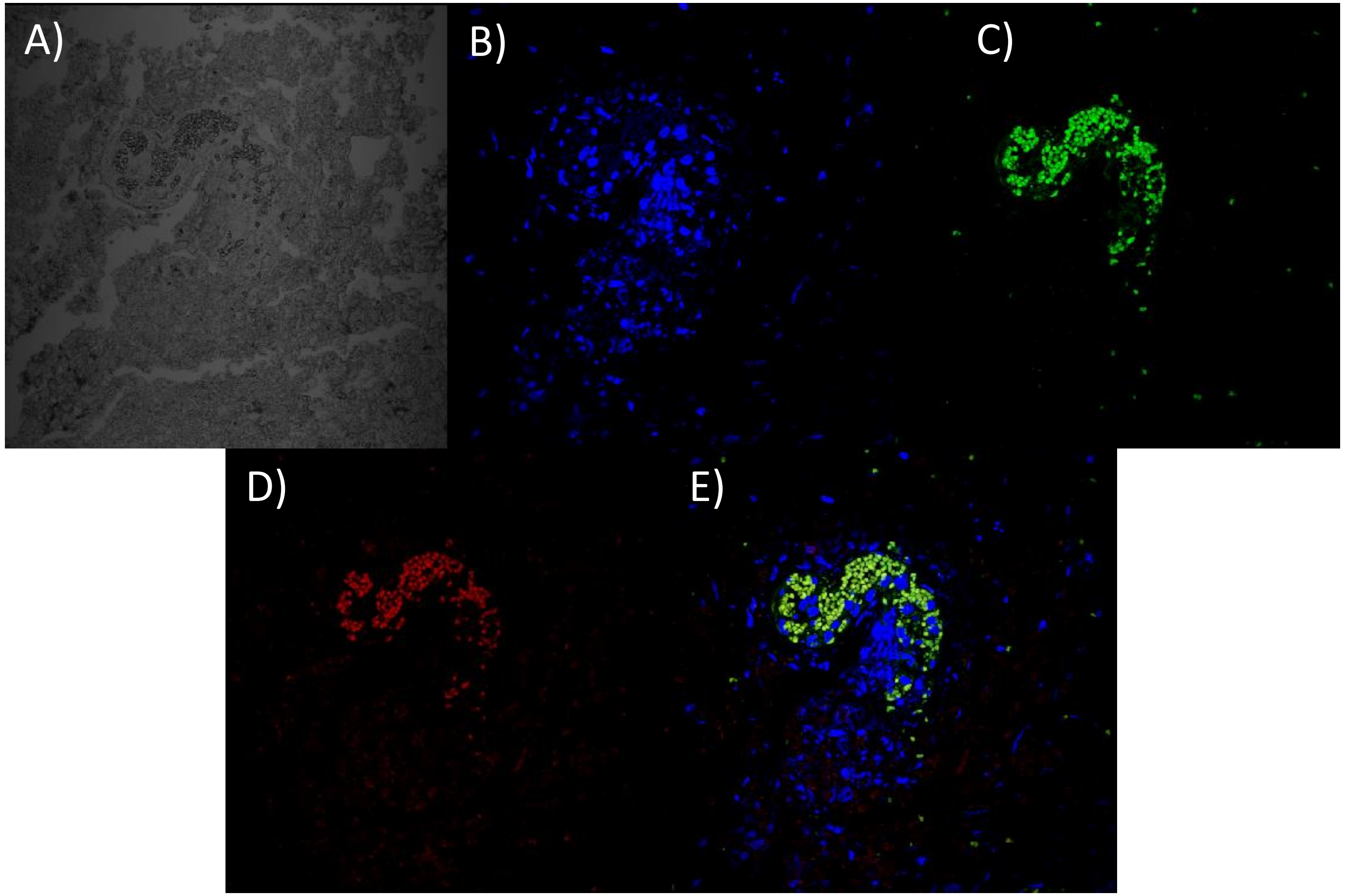

Supplementary Figure S2. Cell staining of NK cells in melanoma sections. A) Without fluorescence. B) DAPI-stained nuclei. C) CD16/32. D) CD56. E) Merged.

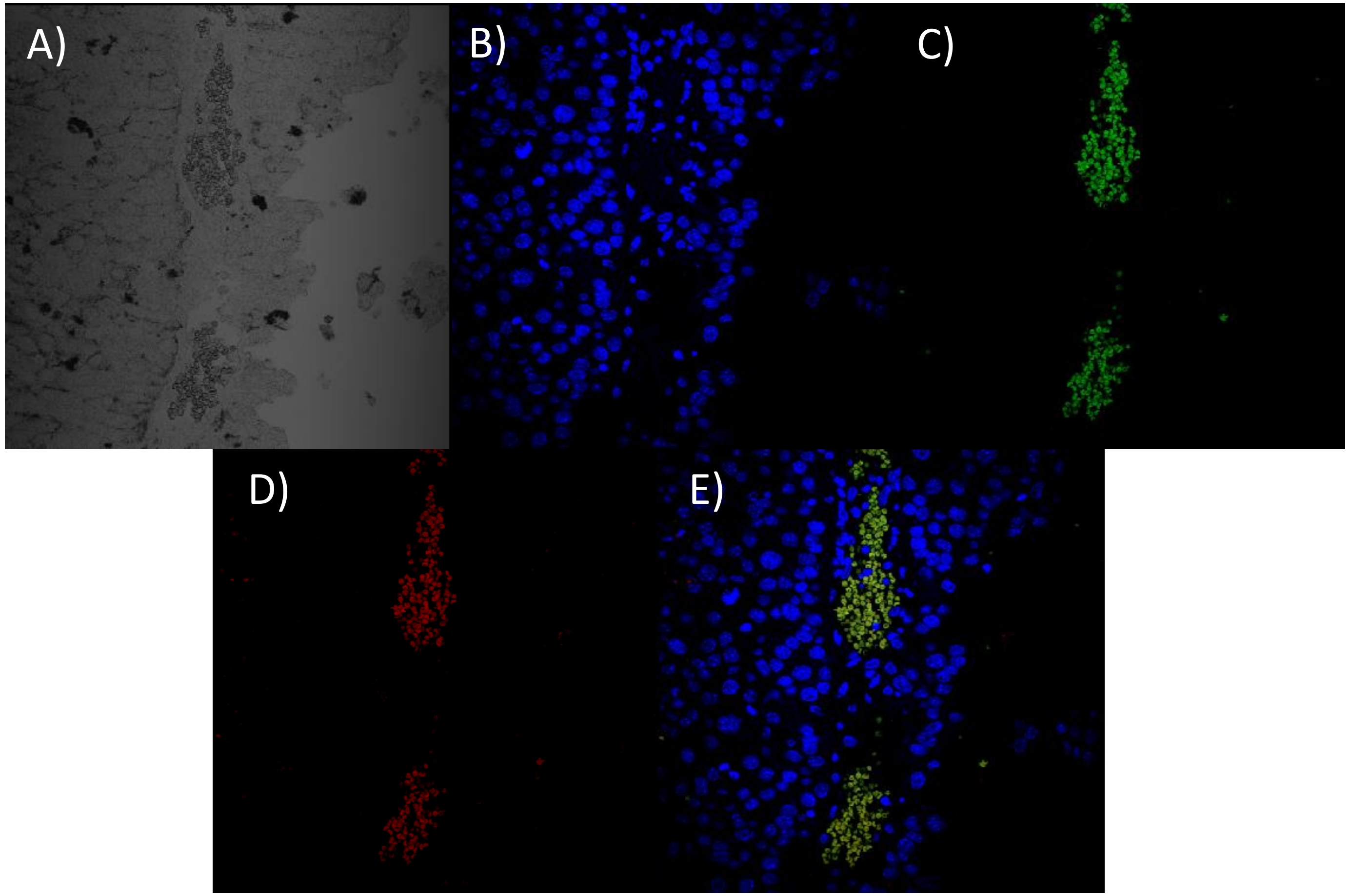

Supplementary Figure S3. Localization of iNKT cells in melanoma sections. A) No fluorescence. B) DAPI-stained nuclei. C) TCRVB8.1. D) CD1D. E) Merged.
